# Formation of Amphiphilic Molecules from the Most Common Marine Polysaccharides, toward a Sustainable Alternative?

**DOI:** 10.3390/molecules26154445

**Published:** 2021-07-23

**Authors:** Tiphaine Wong, Lorette Brault, Eric Gasparotto, Romuald Vallée, Pierre-Yves Morvan, Vincent Ferrières, Caroline Nugier-Chauvin

**Affiliations:** 1 Ecole Nationale Supérieure de Chimie de Rennes, CNRS, ISCR-UMR 6226, Université Rennes, F-35700 Rennes, France; tiphaine.wong@ensc-rennes.fr (T.W.); lorette.brault@gmail.com (L.B.); 2 CODIF, 70 Rue du Commandant l’Herminier, CS 11781, F-35417 Saint-Malo Cedex, France; e.gasparotto@codif.com (E.G.); r.vallee@codif.com (R.V.); py.morvan@codif.com (P.-Y.M.)

**Keywords:** marine polysaccharides, structural modifications, acylation, alkylation, amidation, sustainable processes

## Abstract

Marine polysaccharides are part of the huge seaweeds resources and present many applications for several industries. In order to widen their potential as additives or bioactive compounds, some structural modifications have been studied. Among them, simple hydrophobization reactions have been developed in order to yield to grafted polysaccharides bearing acyl-, aryl-, alkyl-, and alkenyl-groups or fatty acid chains. The resulting polymers are able to present modified physicochemical and/or biological properties of interest in the current pharmaceutical, cosmetics, or food fields. This review covers the chemical structures of the main marine polysaccharides, and then focuses on their structural modifications, and especially on hydrophobization reactions mainly esterification, acylation, alkylation, amidation, or even cross-linking reaction on native hydroxyl-, amine, or carboxylic acid functions. Finally, the question of the necessary requirement for more sustainable processes around these structural modulations of marine polysaccharides is addressed, considering the development of greener technologies applied to traditional polysaccharides.

## 1. Introduction

Major polysaccharides studied from marine resources are extracted from several sources: red, brown, green algae, or seafood wastes. In 2018, the Food and Agriculture Organization (FAO) Globefish Research Program has estimated the global seaweeds market to USD 6 billion per year (approximately 12 million tons per annum in volume). Indeed, around 221 species of seaweed are commercially available and cultivated for human needs. In order to add value to these widely available resources, many structural modulations were proposed so that the resulting application domains are widened. The reactions performed range from depolymerization to total, partial, or selective modifications, comprising acylation, alkylation, and sulfation, to cite the most common opportunities. All these compounds were then studied for instance for their potential in food, detergency, energy, human and plant health, and materials science. The challenges to be overcome are linked to the polymeric nature of the resources and to its variability. Indeed, a unique polysaccharide structure and dispersion is highly dependent on harvest, extraction procedures and also their biosourcing [1,2], resulting in (1) a continuing requirement for adaptation of reaction conditions and, to some extent, (2) properties with some variability. Nevertheless, some simple reactions have been consolidated and will be described here. After a first chapter dedicated to the chemical structures of the main marine polysaccharides, this review focuses on their structural modifications, and especially on hydrophobization reactions which afford products made from those major bioresources, with an emphasis on how the related processes can be improved considering the growing requirement of lowering negative environmental impacts.

## 2. Classes of the Most Common Marine Polysaccharides

### 2.1. Red Algae Polysaccharides

Red algae polysaccharides represent the most important part of the seaweed market. Among them, carrageenans and agar are largely used in food industry and, in a lesser way, extend to the pharmaceutical and cosmeceutical industries. The major red algae species cultivated are *Porphyra*, *Euchema*, *Gracilaria* species, and *Kappaphycus alvarezii* [3]. Polysaccharides extracted are mainly carrageenans and agarans.

**Figure 1:**
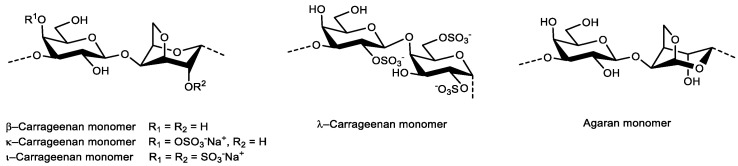
Red algae most representative units.

Carrageenans are present in red algae cell walls [4] and characterized as sulfated polysaccharides presenting an alternance of β-(1,3)-linked-D-galactopyranosyl (G) and α-(1,4)-linked (3,6)-anhydro-D-galactopyranosyl (DA) units (Figure 1). Several types of carrageenans have been identified regarding their sulfation pattern and the number of 3,6-anhydro units. It is worth noting that λ-carrageenan does not present any DA units [5]. Those structural parameters, as well as the presence of sodium and potassium ions in the solution, control the solubility and gelation properties of carrageenans [6,7,8,9].

Agarans differ from carrageenans by the configuration of their 3,6-anhydrogalactopyranosyl entity and the absence of sulfate ester on their backbone. They are made of alternative units of β-(1,3)-linked-D-galactopyranosyl (G) and α-(1,4)-linked (3,6)-anhydro-L-galactopyranosyl units (LA) (Figure 1) [10].

### 2.2. Brown Algae Polysaccharides

*Saccharina japonica*, *Undaria pinnatifida* and *Sargassum fusiforme* are the major brown algae cultivated for commercial uses [3]. They represent, with green species algae, an important food resource in Asia, but they are also used for their biological properties in medical research [11,12,13,14,15]. Among the brown algae polysaccharides, fucoidans, laminarans, and alginic acids are the most notable components. Laminarans and fucoidans are main-water soluble polysaccharides while alginic acids are an alkali-soluble one [16].

**Figure 2:**
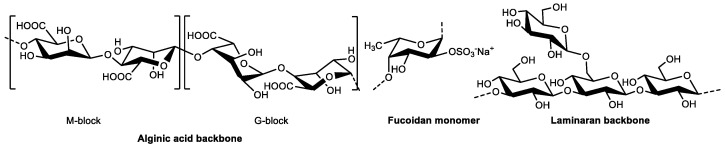
Brown algae most representative units.

The latter are linear block-copolymer of β-D-mannuronic (M unit) and α-L-guluronic acid (G unit), linked by (1,4) glycosidic bonds (Figure 2) [17]. They are arranged in homopolymeric MM, GG blocks, or alternating MG blocks [18]. Alginate (Alg) refers to the sodium alkali form of alginic acid, such as carrageenan and agar polysaccharides, and is a water-gel forming polysaccharide in the presence of multivalent cations, such as Ca^2+^ [19]. Moreover, the M/G ratio and the presence of divalent cations govern the gelling properties of alginate. Thus, higher proportion of G blocks in alginate structure leads to soft and elastic gels [20,21].

Fucoidans are highly sulfated polysaccharides mainly built up of (1,3) and (1,4)-linked α-L-fucose residues (Figure 2) [16]. They also contain other branched structures such as mannose, glucose, galactose, and xylose [11,22]. This variety leads to complex composition and structure elaboration of fucoidans.

Finally, laminarans are composed of β-(1,3)-D-glucopyranosyl units branched with possible β-(1,6)-linked-D-glucopyranose (Figure 2). This last unit determines the water solubility of the polysaccharide. Indeed, increase branching is associated with elevated solubility in water [22]. Among laminarins, two types of polysaccharides are considered regarding their reducing end in the polymer chain. The glucose type only contains glucopyranosyl residues while the mannitol type is ending with 1-*O*-linked D-mannitol [23,24,25].

### 2.3. Green Algae Polysaccharides

*Enteromorpha chlatrata*, now renamed *Ulva chlathrata*, *Monostroma nitidum*, and *Caulerpa spp.*, are the major culti1vated green algae species, according to the FAO Globefish Research Program report of 2018 [3]. Polysaccharides extracted from green algae are less researched than others for their industrial applications. They also present a more varying structure than brown or red algae, which make a general structure more difficult to present. However, it is assumed that green algae polysaccharides are classified under two families: uronic acid rich and uronic acid limited polysaccharides [4].

**Figure 3:**
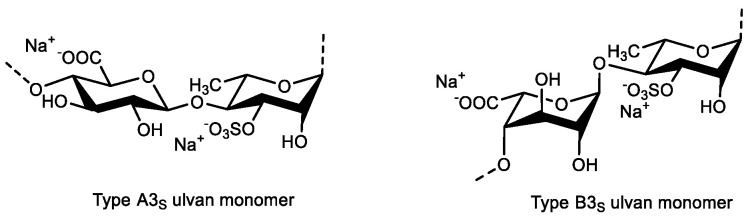
Green algae most representative disaccharidic units found in ulvans.

Ulvans are part of the uronic rich sulfated polysaccharides, and are the most known of green algae polysaccharides. They are usually found and characterized from *Ulva*, *Gayralia*, and *Monostroma* species. However, in 2007, Lahaye and Robic reported that the two main disaccharides units found in ulvans are type A ulvanobiuronic acid 3-sulfate (A3_S_) and type B ulvanobiuronic acid 3-sulfate (B3_S_) (Figure 3) [26]. Those two units differ by the presence of D-glucuronic acid (A3_S_) or L-iduronic acid (B3_S_) linked to the L-rhamnose-3-sulfate. Moreover, different monosaccharides have been found in their composition: glucuronic acid, rhamnose, xylose, and iduronic acid [27,28]. Those monosaccharides are part of the backbone and glucuronic acid is also present in the side chains compositions.

### 2.4. Seafood Wastes’ Polysaccharides

Seafood wastes, such as crustacean shell and shellfish wastes, are sources of chitin polysaccharides. Those polysaccharides are almost as abundant as cellulose, making them an economical raw material of choice. For several decades now, chitin and its derivative have been used, especially in the biomaterial field [29,30,31].

**Figure 4:**
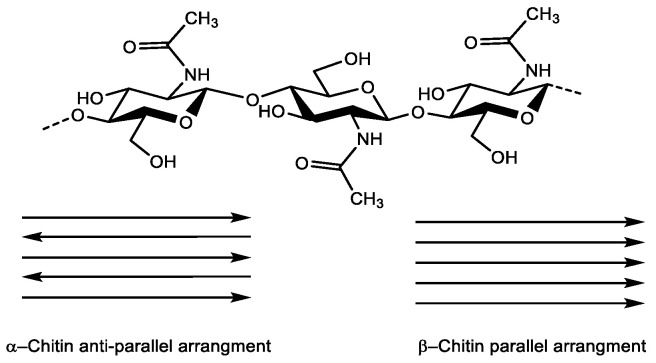
Chemical and crystallinity structures of chitin.

Chitin is a linear polysaccharide composed of β-(1,4)-linked-*N*-acetyl-2-amino-2-deoxy-D-glucosyl (AcAG) units. It presents a poor solubility in water and a high crystallinity [32]. This last parameter induces the differentiation of several type of chitin, the major ones being α- and β-chitin [33]. α-Chitin is the most abundant form and can be found in crustacean; while β-chitin can be found in squid [34]. Those two types of chitin differ from their structures. α-Chitin presents an antiparallel arrangement of the chains, which makes it a more densely packed material. By contrast, β-chitin presents a parallel arrangement [32,34,35]. This last parameter is the reason why β-chitin swells considerably in organic solvents such as methanol, compared to α-chitin (Figure 4) [36,37,38].

**Scheme 1:**
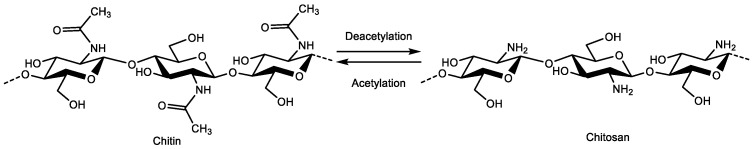
Synthesis of chitosan from chitin.

Chitosan derives from chitin after a deacetylation reaction under basic conditions, and therefore is built up with β-(1,4)-linked-2-amino-2-deoxy-D-glucosyl (AG) (Scheme 1). Chitosan is only soluble in acidic aqueous media as it exhibits the cationic characteristic of the amino groups.

Because of the various sources of chitosan, it is important to characterize it in terms of deacetylation degree (DD) and acetylation degree (DA) [39]. DD and DA measure the fraction of AG and AcAG residues and are evaluated as following (Equation (1)) [40,41]. Most of commercial chitosan presents an average DD value between 70–90%.

(1) $$\begin{array}{c} \mathrm{DD}\%=\frac{n_{\mathrm{AG}}}{n_{\mathrm{AG}}+n_{\mathrm{AcAG}}}\times 100\\ \mathrm{DA}\%=100-\mathrm{DD} \end{array}$$

Calculation of acetylation and deacetylation degree of chitosan.

## 3. Amphiphilic Modifications of Polysaccharides

Chemical modifications of those marine polysaccharides obviously impact their physicochemical and/or biological properties. The major properties of these native polysaccharides are summarized in the Table 1. As an illustration, some interesting chemical modulations as well as the application field of the obtained derivatives are presented for each case. Most of the time, determination of the degree of derivatization was performed using NMR spectroscopy [42], Raman spectroscopy [43], conductimetry titration method [44], and/or elemental analysis [45].

### 3.1. Uronic Rich Polysaccharides

#### 3.1.1. Modification of Carboxylic Groups on Uronic Rich Polysaccharides

Carboxylic acids present on uronic acid rich polysaccharides, such as alginate or ulvans, can be targeted for amphiphilic modifications of polysaccharides. Those modifications can be classified under three sections: esterification, amidation, or modulation by an Ugi reaction.

- Esterification

**Scheme 2:**
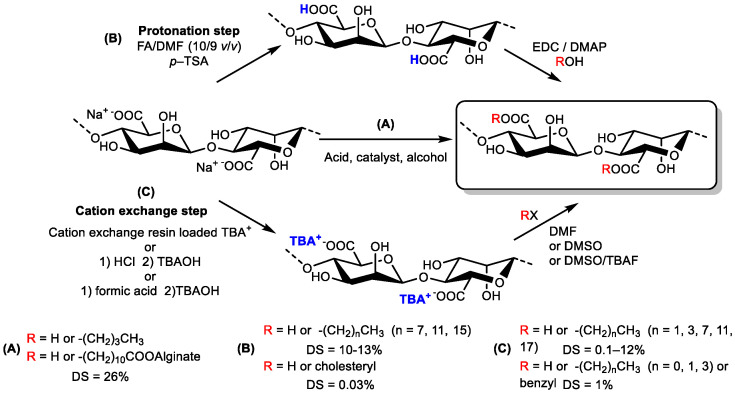
Esterification procedure on alginate carboxylic groups.

The standard Fisher esterification reaction requires acidic conditions in order to protonate the carboxyl group, promoting the grafting of chosen alcohols. Thus, reaction conditions could also promote partial depolymerisation through acido-catalyzed breaking of glycosidic linkages. Usually, the catalysts used for this modification are sulfuric acid [64] or *para*-toluenesulfonic acid (*p*TSA) [65]. In 2006, Broderick et al. modified the carboxylic function of alginate using sulfuric acid as catalyst and butanol as reactant and solvent [64]. The advantages of this pathway were the mild conditions and the minimization of the number of reactants. In 2013, Wu et al. have formed cross-linked alginate using 1,10-dodecanediol in a *p*TSA/dimethylformamide (DMF) media (Scheme 2A) [65]. However, most esterifications of alginates occurred after a first step of protonation of sodium alginate to obtain the alginic acid. For that, sodium alginate was stirred in a mixture of formamide (FA)/DMF (typically 10/9 *v*/*v*) in presence of *p*-TSA. After the protonation step, the alginic acid was reacted with the chosen alcohol in presence of 4-dimethylaminopyridine (DMAP) and carbodiimide (dicyclohexylcarbodiimide (DCC) or 1-ethyl-3-(3-dimethylaminopropyl) carbodiimide (EDC)). Such a procedure was used to graft dodecanol [19], hexanol, octanol [66] and cholesterol [67] on alginates (Scheme 2B). A last possibility for esterification is to perform a S_N_2 nucleophilic substitution reaction, with alkyl halides. This was performed using tetrabutylammonium (TBA)-alginate. The use of TBA as counter-cation of the carboxylate group allowed a better solubility of polysaccharide in organic solvent thanks to the aliphatic residues. This artifice was firstly described by Della Valle et al. in 1990, for hyaluronic acid modification, using a cation exchange resin loaded with TBA [68]. A few years later, Babak et al. adjusted that protocol by replacing the direct ion exchange by an heterogeneous acidification with HCl of Na-Alg followed by neutralization with tetrabutylammonium hydroxide (TBAOH) [69]. Some other groups chose to acidify alginate with formic acid, before neutralization with TBAOH [70]. When performing a S_N_2 reaction, the use of anhydrous conditions is an important parameter as water could also act as the nucleophile. Such an S_N_2 procedure was performed to graft dodecyl [69], octadecyl [71], ethyl [72], and butyl [72] chains on alginate (Scheme 2C). The choice of the solvent for this procedure was also important. Indeed, DMSO has been shown to react with alkyl halides, leading to alkyloxydimethylsulfonium halide formation. Because of that side reaction, DMF was established as more suitable while using halide alkyl donors [72].

- Amidation

**Scheme 3:**
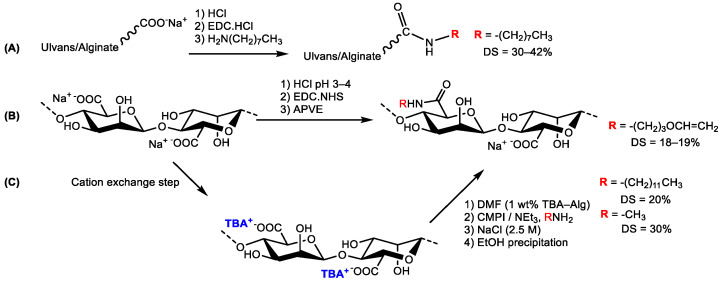
Amidation procedures on ulvans and alginates.

Amidation on the carboxylic group is another way to obtain hydrophobized compounds from uronic acid-containing polysaccharides. This reaction is performed in acidic aqueous media (pH 3–4) to ensure the presence of the carboxylic acid. The carboxylic acid is then activated by adding a carbodiimide coupling agent. This modification was firstly developed for alginates [73,74,75,76] and further adapted by Sari et al. for ulvans to graft octylamine, using the EDC.HCl coupling agent (Scheme 3A). Conjugated alginate was also grafted with an aminopropyl vinyl ether (APVE) using EDC.NHS (*N*-hydroxysuccinimide) as coupling agent [77] (Scheme 3B). Other aminoalkylchains were used to modify alginate, such as dodecylamine [78] or methylamine [79]. Those later modifications were performed using TBA-Alg form. The amidation was then performed using CPMI (1-chloro-1-methylpyridinium iodide) and triethylamine (Scheme 3C).

In a very interesting approach, Fort et al. introduced lipophilic amino acids through amidation of the carboxylic functions of oligoalginates [57]. The grafting reactions were conducted with the 4-(4,6-dimethoxy-1,3,5-triazin-2-yl)-4-methylmorpholinium chloride (DMTMM) coupling agent which is compatible with non-protected hydroxyl groups and is less prone to formation of by-products. The amidation reactions were performed starting with the methyl esters of alanine or leucine, followed by a saponification to afford the desired conjugates in 70–93% yields. The authors demonstrated that such a structural modulation gave more resistant or non-affected oligosaccharides toward the hydrolytic activity of alginate lyases.

- Ugi reaction

**Scheme 4:**
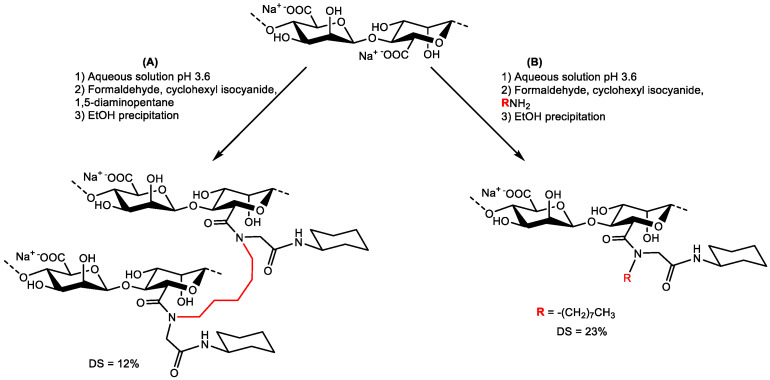
Ugi condensation on alginates.

Finally, carboxylic acids of alginates and ulvans can undergo a multi-component Ugi reaction. An imine, obtained by condensation of a primary amine with an aldehyde or a ketone, is protonated by the carboxylic acid. Then, a cascade of two additions followed by an intramolecular rearrangement yield a bis-amide compound. This kind of modification, at the best of our knowledge, has only been performed on alginates. To perform this condensation reaction, one of the important parameters is the pH of the reaction, which should be set at 3.6. The advantages of this reaction are the high yield obtained and the fact that almost all of the atoms involved in the reaction are used to obtain the final compound, thus addressing the question of atom economy. However, the components, except for the alkylamine, are usually added with 40% excess. In 2004, Bu et al. performed Ugi condensation on alginate to obtain a cross-linked alginate with a pentyl spacer (Scheme 4A) [80]. More recently, in 2016 and 2018, Yan et al. [81] and Zhao et al. [82] synthetized an Ugi modified alginate in presence of formaldehyde, cyclohexyl isocyanide, and octylamine (Scheme 4B).

#### 3.1.2. Modification of Hydroxyl Groups on Uronic Acids Rich Polysaccharides

Alginic acids and ulvans present two hydroxyl functions on C-2 and C-3. Those positions have been subjected to modification via esterification reactions. Moreover, considering the ability of these polysaccharides to form various gels, the reactivity on those hydroxyl groups is highly dependent on the polysaccharide solubility and thus on the hydroxyl groups availability. Finally, specific modifications of either hydroxyl on C-2 or C-3 positions were difficult to control as their reactivities were very similar.

**Scheme 5:**
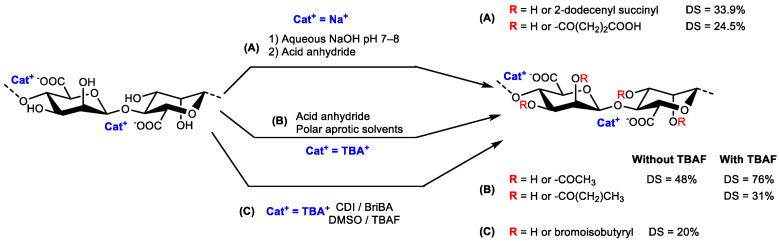
Alginate hydroxyl modifications.

Hydroxyl modifications of alginates were inspired from protocols of modifications of starch, amylose, and amylopectins published in 1951 by Wolff et al. and adjusted by Phillips et al. in 2000 [43,83]. As alginic acids were only alkali-water soluble, the hydroxyl modifications were usually performed in an aqueous media, using Na-alginate salts. Thus, for esterification of alginate, the pH of the reaction media played an important role in the esterification reaction and was kept between 7 and 8 by addition of NaOH solution. Examples found in literature explored the grafting of short succinyl-like chain using succinic anhydride [84] and 2-dodecenyl succinic anhydride [85,86,87]. Compared to carboxylic modifications, those works open pathways for greener synthetic alternatives of amphiphilic alginates as they occurred in aqueous media (Scheme 5A). Nevertheless, some reactions were performed in polar aprotic solvent, such as DMSO or DMF. To achieve solubilization of alginate, TBA (tetrabutylammonium)-alginate was used. Then, a dissolution promoter, such as tetrabutylammonium fluoride (TBAF) could also be added to perform a total solubilisation of the TBA-alginate form. Such an example is found in the work of Pawar et al. who modified the hydroxyl groups of alginates using a similar procedure as for carboxylic acid modifications. They showed that addition of TBAF (10% *w*/*v*) allowed a complete solubility of the polysaccharide in polar aprotic solvent while it is only partially dissolved without TBAF [88]. As a consequence, substitution degrees were higher in homogeneous solutions where TBAF was added. However, this workers’ group only grafted short carboxylic acid chains, such as acetic or propionic, and the use of hexanoic anhydride showed no grafting. They explained this last result by the competition between the hydrophobic nature of hexanoic anhydride and the polycharged nature of the TBA-alginate [88]. In their study, mixture of TBAF with solvent such as DMSO, DMF, DMAc (dimethylacetamide), and DMI (1,3-dimethyl-2-imidazolidinone) were also studied and allowed a total solubilization of TBA-alginate salts (Scheme 5B). In the same register, in 2015, Kapishon et al. grafted bromoisobutyryl chain using TBA-alginate salts dissolved in 2% *w*/*v* TBAF containing DMSO, in the presence of 1,1’-carbonyldiimidazole/α-bromoisobutyric acid (CDI/BriBA) [89]. CDI was used as an esterification activator for grafting of α-bromoisobutyryl chain (Scheme 5C).

**Scheme 6:**
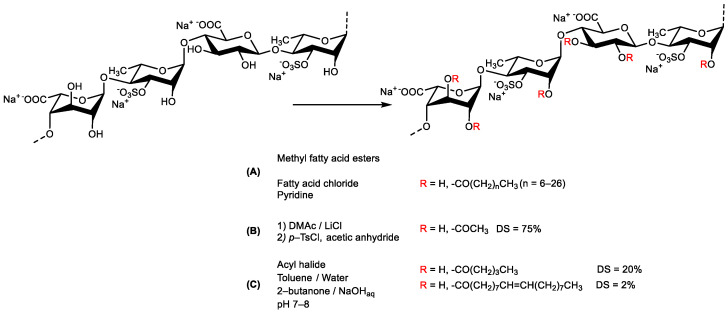
Ulvan hydroxyl modifications.

Direct-nhj jkugiu-iyoi modifications of ulvans with fatty acid chains varying from C_8_ to C_28_ was patented by Ranson et al., in 2006 [61]. For that, hydroxyl modifications were usually performed by esterification or transesterification reactions. For esterification, ulvans were modified in pyridine, using fatty acid chlorides at 130 °C during 2 h. Ulvan esters were isolated by precipitation and different steps of washing. Transesterifications were mostly performed without solvent, using fatty acid esters, and especially methyl fatty acid esters at 150 °C for 6 h. Final products were recovered by dissolving the reaction media in butanone followed by a neutralization with lactic acid to obtain a gel product. During that procedure, degradation of ulvan chains was observed, resulting in a coloration of the final product. (Scheme 6A). In 2012, Qi et al. acetylated ulvans [60] adapting the protocol developed by Tosh et al. for the acetylation of cellulose [90]. Ulvan polysaccharides were thus firstly dissolved overnight in DMAc using LiCl. Then, the mixture was diluted to 1 wt%, *p*-TsCl was added with acetic anhydride and the reaction was performed at 60 °C for 10 h to give acetylated ulvans (Scheme 6B). Finally, in 2019, Morelli et al. extended their protocol from acrylated ulvan synthesis [58] to prepare ulvan esters with butyryl and oleyl acid chains [59]. They carried out the modification in a biphasic mixture of water and toluene and added a compatibilizer solvent: 2-butanone which was firstly pre-activated by addition of NaOH. Acyl halide was added in a large excess to compensate the loss due to its hydrolysis in aqueous media. The reaction was performed at 4 °C, pH 7–8 for 3 h (Scheme 6C).

### 3.2. Aminated Polysaccharides

Aminated polysaccharides include chitin and its derivative chitosan, which are a resource of choice as they are non-toxic and biodegradable polymers [45]. However, most of the modifications listed from literature are carried out on chitosan, which, in addition to the C-3 and C-6 hydroxyl groups, also presents a C-2 primary amino-group, unlike chitin. Hence, it is an advantageous polymer for long acyl chain grafting to yield new amphiphilic structures. Moreover, chitosan is insoluble in most of the common organic solvents used for hydrophobization reactions, but it exists partially as a quaternary ammonium salt in aqueous acidic solution of acetic, formic, propionic, butyric acid, etc, allowing its solubility [91].

#### 3.2.1. Amino-Modifications

Since an amine function is significantly more nucleophilic than a hydroxyl group, modifications of chitosan are mostly performed via this function. *N*-alkylations, reductive amination and *N*-acylations are the main ways to obtain amphiphilic molecules. Moreover, amino groups can also be modified to quaternary amino chains leading to amphiphilic polymeric chitosan derivatives, as carriers for hydrophobic bioactive molecules [62].

**Scheme 7:**
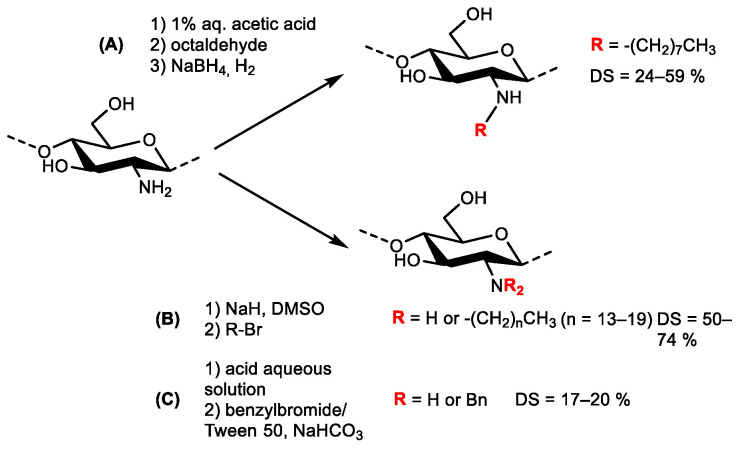
*N*-Alkylation procedures on chitosan.

- *N*-Alkylation;

At the beginning, *N*-alkylation of chitosan was performed by reductive amination. This procedure was previously described by Yalpani and Hall in 1984 [92] and Holme et al. in 1986 [93]. They used NaBH_3_CN as reductive agent in a methanolic solution containing 1 v% of aqueous acetic acid. Later on, similar procedures were described using different reductive agents. One example was described by Huo et al. in 2010, to introduce an octyl graft from octaldehyde and using NaBH_4_ in aqueous solution as reductive agent under hydrogen (Scheme 7A) [94]. *N*-isopropyl chitosan was described in Cok and coworkers’ work using acetone and picolidine-borane complex as reductive agent in a methanol/aqueous acetic acid solvent [95]. By varying the molar ratio of acetone, they managed to obtain different isopropyl derivatives. Direct alkylation was also described, for example, by Zhou et al. in 2011. In this work, they described a two-step alkylation, addition of NaH in DMSO followed by nucleophilic substitution of *n*-alkyl bromide (Scheme 7B) [96]. In 2012, Kurita and coworkers described alkylation performed in acidic aqueous solution using sodium hydrogen carbonate and a mixture of alkyl halide/Tween 50 during a 24 h reaction at 80 °C [45]. Tween 50 was added to disperse homogeneously the alkyl halide in the media. The use of NaHCO_3_ instead of NaH brought the modification to an environmentally friendly process as it was a less toxic reagent (Scheme 7C).

**Scheme 8:**
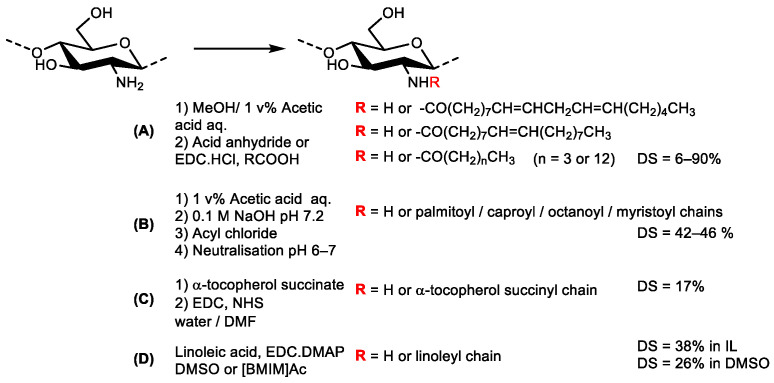
Some of the *N*-acylation procedures on chitosan.

- *N*-Acylation;

Like *N*-alkylations, most *N*-acylations were performed using aqueous acetic acid solution mixed with methanol or ethanol for a better solubility of chitosan. Several examples can be identified from literature, such as grafting of acyl chain from linoleic acid, oleic acid [44,97,98], myristoyl anhydride [99], or caproic acid [100]. While using the carboxylic acid, EDC.HCl, sometimes coupled with *N*-hydroxysuccinimide (NHS), was used as a coupling agent. However, the use of the corresponding anhydrides prevented the addition of further catalysts (Scheme 8A). While using fatty acyl chloride, the acylation was performed in aqueous solution at pH 6–7 without the addition of any catalyst or co-solvent, simplifying the procedure. Such procedure was described in 2003 by Le Tien and co-workers, who grafted fatty acid chains from C_6_ to C_16_ on chitosan (Scheme 8B) [84]. DMF was sometimes used as co-solvent in water to homogenize the reaction medium. This procedure was described in the work of Liang and co-workers where α-tocopherol succinate-modified chitosan was synthetized. The resulting conjugated polysaccharide found application as micellar delivery system for paclitaxel (Scheme 8C) [63]. Fewer works have described *N*-acylations in organic solvents monophasic systems. In 2006, Vasnev and co-workers prepared acylated chitosan using different conditions. One of them was the use of DMAc as solvent and a pre-treatment of chitosan in an aqueous solution of trifluoroacetic acid. After water removal, myristoylation with the acyl chloride was performed in the presence of pyridine and triethylamine (TEA).The authors also described a procedure using DMAc-LiCl solution using *p*-nitrobenzoyl chloride [101]. More recently, the emergence of ionic liquids (IL) allowed *N*-acylations of chitosan. Argüelles-Monal et al. compiled those works in a review in 2018 [102]. In brief, imidazolium-based ionic liquid was described as one of the most efficient IL as it also acts as an excellent catalytic medium [103]. For example, linoleic acid chain was grafted on chitosan using linoleic acid, EDC as coupling agent, DMAP as nucleophilic catalyst and 1-butyl-3-methylimidazolium acetate ([BMIM]Ac) or DMSO as solvent [104]. As a result, they showed that the use of [BMIM]Ac gave better yield and DS value compared to the same reaction performed in DMSO (Scheme 8D). Moreover, they also showed that the IL could be reused for at least eight cycles without any change in its structure.

- Quaternization of Chitosan

**Scheme 9:**
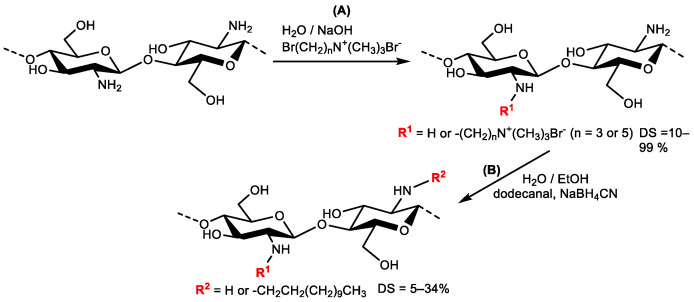
Quaternization procedures of chitosan.

Quaternization of chitosan is another modification that can be performed on their amino-group. Formation of such amphiphilic molecule allowed chitosan to have a densified positive charge and thus increased its solubility at higher pH [105]. Those modifications were usually performed in NaOH aqueous solutions using quaternary ammonium bromides (Scheme 9A) [105,106,107]. The obtained compounds were further modified by reductive amination using alkyl aldehydes and NaBH_3_CN (Scheme 9B) in order to mask the remaining free amino groups.

#### 3.2.2. Hydroxyl Modifications

**Scheme 10:**
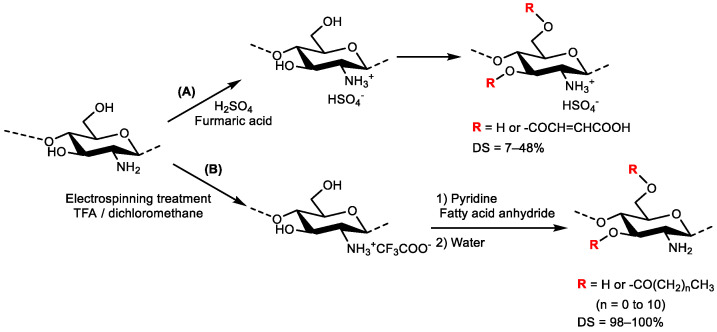
Acylation procedures of chitosan.

Besides the amino function, two hydroxyls groups at C-6 and C-3 offer subsequent opportunities for chemical modulations. Theoretically, *N*-protection is required before *O*-acylation or alkylation. Nevertheless, inversion of reactivity was interestingly and efficiently performed under acidic conditions which generate non-nucleophilic ammonium salts. Trifluoroacetic acid (TFA), methanesulfonic acid, or sulfuric acid were usually proposed to meet this reactivity [108]. In this context, Feng and co-workers described in 2011 a specific *O*-acylation of chitosan by fumaric acid in presence of H_2_SO_4_ during 4 h at 80 °C (Scheme 10A) [109]. Later on, in 2017, Zhang and co-workers described a two-step procedure which began with a pre-treatment of chitosan with TFA in dichloromethane and an electrospinning treatment to form chitosan nanofiber membranes. The latter were subsequently *O*-acylated in a pyridine/carboxylic acid anhydride solution for 2 h at 80 °C (Scheme 10B) [110].

### 3.3. Sulfated Polysaccharides

Fucoidans and carrageenans are sulfated polysaccharides which can be modified on their different hydroxyl groups to obtain amphiphilic molecules [111]. However, to our knowledge, those polysaccharides were the object of only few studies which mostly concerned the grafting of acyl chains on hydroxyl positions. In their review published in 2009, Campo and coworkers listed the different procedures to esterify carrageenans [5]. Since then, no recent work has been found on grafted carrageenan or fucoidan procedures.

Such as for alginates and ulvans, carrageenans modifications were performed using the sulfate-TBA salts which triggered the solubility in the most commonly used organic solvent, such as DMF. Thus, conditions of acylation were classical and inspired from Petitou et al. [112], where heparin sulfate was *O*-acylated using TBA salts.

**Scheme 11:**
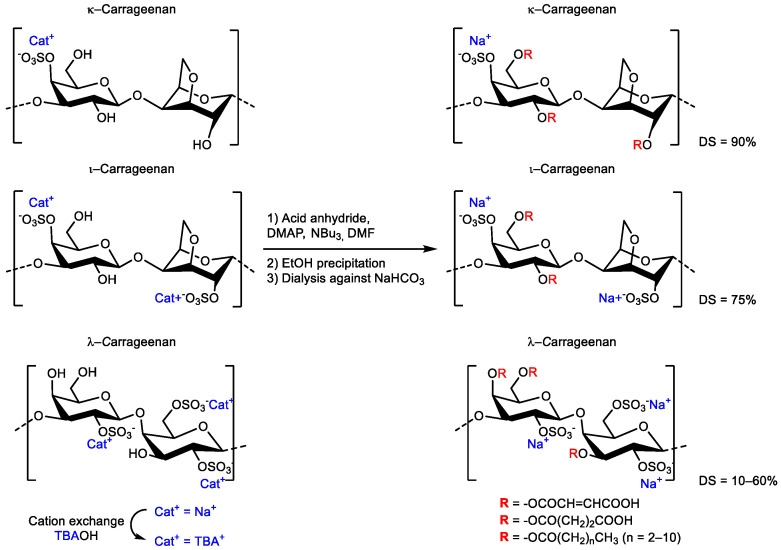
Acylation of carrageenans.

Another issue with the modification of carrageenans was the presence of the (3,6)-anhydrogalactopyranosyl units which narrowed the range of conditions because of its acid-labile sensibility [113]. Then, acylation reactions were performed using DMAP as catalyst, tributylamine as base for pH control, and carboxylic acid anhydride as acylating agent. Nowadays, only two major groups have been working on commercially available κ-, ι- and λ- poly- and oligocarrageenans for modifications by grafting fatty acid with length chain varying from C_4_ to C_12_ (Scheme 11) [42,46,47,48,49]. It is noteworthy that the degree of substitution could reach 90% under these conditions.

**Scheme 12:**
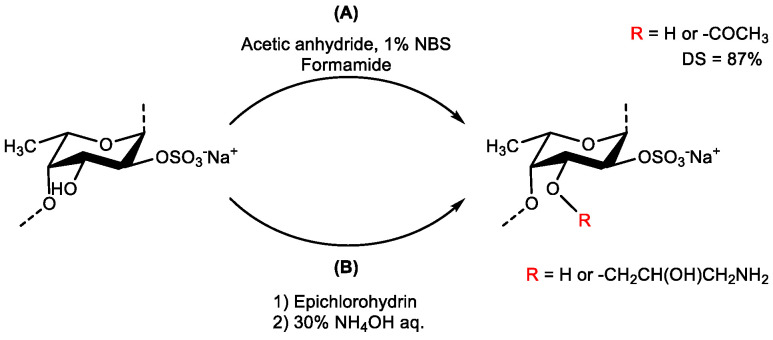
Acylation of fucoidans.

Even if the hydrophobization of fucoidans is still underexplored, a procedure involving acetic anhydride, formamide, and 1% of *N*-bromosuccinimide (NBS) yielded the desired acetylated fucoidan (Scheme 12A) [53]. An alternative to make this marine resource less polar was introduced by Soeda and coworkers [54,55]. It relied on first introducing a spacer ended with an amino group (Scheme 12B).

### 3.4. Hydroxyl Only Polysaccharides

#### 3.4.1. Agarans’ Modifications

The modification of agarans into hydrophobic compounds is inspired from modifications of starch [50]. However, the difference in the availability of hydroxyl groups between glucose and galactose units makes agarose less reactive than starch, and its gelling properties also complicate its handling. In decreasing order of reactivity, hydroxyl groups of agarans react in the following order: C6-OH > C2-OH > C4-OH [114,115].

- *O*-Alkylation of agarans

Most of the works found in the literature describing alkylation of agarans are based on opening of epoxides and aimed to modulate the absorption and desorption properties of agarose for applications on HPLC. The first study was reported at the end of the 19th century [116,117] using epoxy derivatives as coupling agent in two-step-reactions. More recently, a single-step synthesis has been reported in order to modulate the rheological and thermal properties of agarans under milder reaction conditions [51,52].

**Scheme 13:**
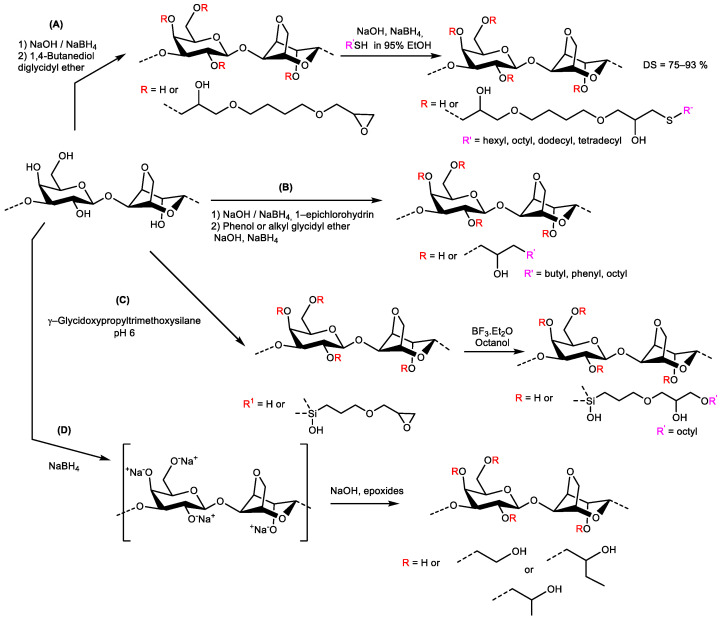
Agaran *O*-alkylation.

The principle of the two-step method is based on the reaction of a bifunctional epoxy precursor (epichlorohydrin [117] or alkyl diglycidyl ether derivatives [116,117]) in the presence of NaOH and NaBH_4_. Then, the remaining epoxy function reacted with an alkyl mercaptan. This procedure was firstly described by Maisano et al. in 1985 for the synthesis of alkyl sulphide derivatives of agarose resin (Sepharose 6B). They used 1,4-butanediol diglycidyl ether as coupling agent and alkyl mercaptan as hydrophobic reagent (Scheme 13A) [116]. A few years later, the synthesis proposed by Oscarsson et al. in 1989 relied on epichlorohydrin as coupling and cross-linking agent, and phenol as hydrophobic reagent (Scheme 13B) [117].

Other methods with different conditions have been reported, including the one of Hjertén et al., which consisted first on an activation of agarose resin (Sepharose 4B) in water with γ-glycidoxypropyltrimethoxysilane followed by the grafting of a fatty alcohol in the presence of boron trifluoride diethyl etherate. This modification is particular by grafting the siloxane group onto two agarose chains, conducting to the reticulation of the polysaccharide (Scheme 13C) [118]. The process was simplified in 2015 and 2018 by Zhang et al. who reported a one-step synthesis of amphiphilic agarose derivatives. The reaction involved epoxides (ethylene oxide, 1,2-propylene oxide, or 1,2-epoxybutane) with agarose in aqueous basic conditions [52]. For the substituted epoxides, the reactions occurred only in the presence of sodium borohydride [51]. However, they reported that the reactivity of those epoxides towards the agarose decreased with the length of the side chain, which then limited the hydrophilic lipophilic balance (HLB) modulation (Scheme 13D).

- *O*-Acylation

**Scheme 14:**
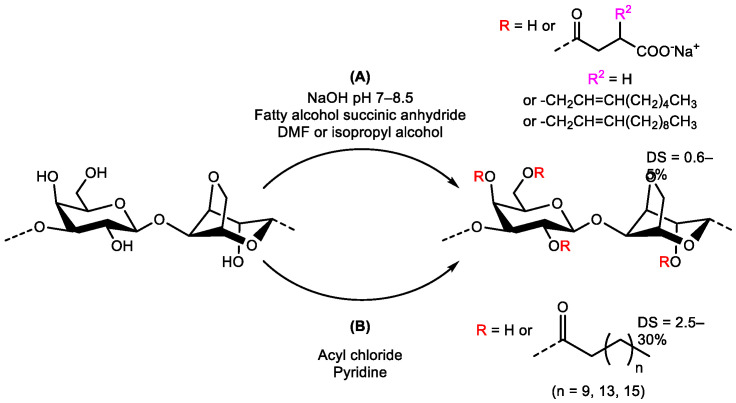
*O*-Acylation of agarose.

Surprisingly, the chemical processes developed for conventional polysaccharides such as cellulose and starch [119], have been only very recently taken up for modifications of agars, as acetylation [120], and later on as acylation with anhydrides and acyl chlorides for the preparation of amphiphilic agarose based molecules [121,122]. This was particularly the work of Xiao et al. in 2019, who first synthesized agarose grafted with octenyl or dodecyl succinic acid. The process consisted of mixing an aqueous solution of agarose (7 wt%, pH 8.5) with a solution of 5% alkenyl succinic anhydride in isopropyl alcohol or in DMF (Scheme 14A). Since the degree of substitution (DS) was low (≈5%), another process was developed using fatty acyl chlorides in pyridine. Those last processes allowed the synthesis of higher substituted fatty esters of agarose with lauroyl, stearoyl, or palmitoyl chains and a DS of about 30% (Scheme 14B).

Although the reactivity of carboxylic acids is lower compared to that of anhydrides and acyl chlorides, their use is a key point for development of a green process. Indeed, unsaturated as well as saturated fatty acids are directly extracted from biomass, and do not require any harsh pretreatment, unlike other conventional acyl donors. In 2005, Prasad et al. began research in this direction and proposed a protocol to access to amphiphilic agars [50]. They used fatty acids such as lauric, myristic, stearic, palmitic, and oleic acids, in methanol without any catalyst. However, even if they showed some interesting variations in the rheological properties of agar gels, the DS were either minor or null. In fact, fatty acids acted as chelating agents between two hydroxyl functions of agars backbones, forming a fatty acid-agar complex but without covalent modification of the polysaccharide.

#### 3.4.2. Laminarans

**Scheme 15:**
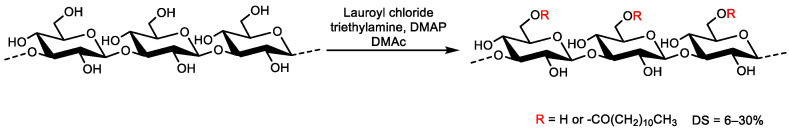
*O*-Acylation of laminarans.

Laminarans are, like agarans, hydroxyl only polysaccharides but do not have any gelling properties. This last parameter facilitates the handling of the polysaccharide and the availability and reactivity of the hydroxyl groups. As at this day, Paris et al. is the only group who have been interested in the hydrophobic modulation of laminarans. The latter were partially grafted with lauroyl chains on the primary OH groups to improve the antimicrobial activity of laminarans against *Plasmorata viticola*. As for *O*-acylation of agarans or ulvans, laminarans were grafted using fatty acyl chloride in a polar aprotic solvent (DMAc) and in the presence of DMAP as catalyst and TEA as acid-scavenger (Scheme 15) [56].

## 4. Tools and Techniques to Develop More Sustainable Processes

**Figure 5:**
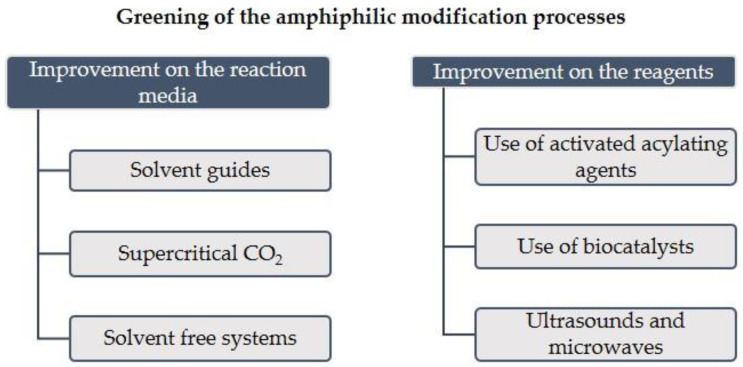
Tools for the development of sustainable routes for amphiphilic polysaccharides.

Amphiphilic polysaccharides are generally used for applications in the pharmaceutical, cosmetics, or food industries. At present, most of the previous synthesis pathway presented above could not reach the “green label”. However, over a few decades, interests have emerged to work on the improvement of the environmental impact of processes around the structural modulations of natural polysaccharides. Indeed, sustainable and cost-effective pre-treatment, extraction, and purification methods are still needed for industrial applications. This attention firstly focused on improving the environmental impact of polysaccharide extraction and purification, using for example supercritical fluids, ultrasounds, or microwaves assisted systems (Figure 5) [123,124]. Moreover, because of the field of application of such molecules, industries have been sensitized to reduce the use of compounds presenting toxic, environmental, or health hazards, by choosing alternative solvents and reagents. So far, most of the attention to this environmental impact focused on modifying traditional polysaccharides, such as hyaluronic acid, cellulose, or starch. Examples of starch modification are presented in Table 2. The application of innovative modification processes to marine polysaccharides through the use of greener technologies (such as ILs, solvent-free systems, etc.) remain rare. A few of them are presented herein.

Since the solvent is the major waste in a process, its choice, if its presence is required, has to be considered very early in the design of a greener process. Structural modifications of marine polysaccharides are already green in some cases. For example, the *N*-alkylation of amino-polysaccharides or *O*-alkylation of agarans already occurred in aqueous media [117]. Moreover, the use of ionic liquids has been tested for *N*-acylation of chitosan. However, several chemical routes remain to be improved, especially the ones where organic solvents such as dichloromethane, DMF, DMAc, or pyridine are used.

An alternative to standard solvents relies on the use of ionic liquids (ILs). The latter are salts with low melting points composed of an organic cation and either an organic or an inorganic anion characterized by a smaller size than the cationic one. They are well known for their low volatility, low inflammability, good thermal and chemical stability, as well as their high ionic conductivity and recyclability. Considering the aforementioned characteristics, ILs can be seen as green solvents. Among them, it is noteworthy that 1-ethyl-3-methyl-imidazolium acetate ([EMIM]OAc) presents a remarkable ability to dissolve crystalline polysaccharides. For example, it was used to dissolve cellulose for heterogeneous [125] or homogeneous [130] modifications while 1-butyl-3-methyl-imidazolium acetate ([BMIM]OAc) was used for *N*-acylation of chitosan [84].

Interests in biocatalysis emerged in the 1990s and were first undertaken for modification of small saccharides [131], such as glucose [132], lactose [133], saccharose [134], maltose, or maltotriose [135]. For a few years, they were also used for modification of conventional polysaccharides, such as cellulose or starch [136,137,138,139]. Aside from replacing the conventional catalyst with a more environmentally friendly one, the use of biocatalysts also allows the development of processes with milder conditions and target modification of polysaccharides with a lower substitution degree leading to different properties for those amphiphilic molecules. Moreover, as biocatalysts are mostly used under mild conditions of temperature or pressure, polymer degradation is less likely to happen, as opposed to other chemical processes. Their regioselectivity also allows a modification at a specific position without any upstream protection steps. Usually, proteases and esterases are used for enzymatic modification of carbohydrate with short fatty acid chains while lipases are favoured for long fatty acid chains. This topic has been discussed in a variety of reviews [128,138,140]. It has been demonstrated that acetylase is part of a protein complex including four proteins encoded in the alginate biosynthetic gene cluster, necessary for alginate acetylation [141]. It belongs to the alginate-modifying enzymes used as tools for alginate characterization. However, engineered enzymes from these natural ones may then be used alone or in chemoenzymatic approaches to create modified polysaccharides with new and desired functionalities.

## 5. Conclusions and Future Outlooks

Many applications are available thanks to hydrophobization of marine polysaccharides. Most of them concern acylation, mainly acetylation, reactions increasing hydrophobicity of polymers likely to modify their physicochemical properties, such as gelling, thickening, and emulsifying. For instance, it has been demonstrated that the degree of acetylation affects the water-binding properties and increases the viscosity of the alginate [141]. These modifications are likely to apply to bioactive compounds with interesting health promoting effects on human or animals. However, likely to the native polysaccharides, the physicochemical properties of the modified ones could be affected by the complex chemical structure, the type and concentration of cations and other compounds (salts, proteins, etc.). Their bioactive properties are susceptible to be modulated by the molecular weight, constituent sugar linkages, and degree of branching. Consequently, the generation of smaller units with better defined molecules could be of interest and more suitable for applications requiring sustainable depolymerization processes. However, considering the rare examples described in the literature, the biocatalyzed esterification of algal polysaccharides remains to be challenged.

The recent developments that meet more and more issues of the 12 rules of the green chemistry, resulted in innovative products and substantial financial savings. Some limitations, however, remain to be overcome in order for marine resources to be structurally modified through more environmentally friendly procedures. Some technologies already applied to the modulation of terrestrial or animal polysaccharides are still to be challenged for the marine counterparts. Moreover, thanks to tremendous increases in basic knowledge in marine enzymes, essentially hydrolases, lyases, (de)sulfatases, and (de)acetylases, in combination with new media and techniques, one can predict great advances for the synthesis of complex but structurally well mastered polysaccharides, for their degradation, and for original structural modulations for obtaining products with very high added value.

## Figures and Tables

**Table 1:** Physicochemical properties of modified polysaccharides.

Polysaccharides (Seaweed Sources)	Physicochemical Properties	Chemical Modifications	Applications of Modified Polysaccharides	Reference
Carrageenans (Red Algae)	Gelling properties (Depending on Na^+^/K^+^ Concentration)	*O*-acylation	HIV treatment	[42,46,47,48,49]
Agarans (Red Algae)	Gelling properties	*O*-alkylation O-acylation	Modulation of rheological and thermal properties/HPLC applications	[50,51,52]
Fucoidans (Brown Algae)	Highly sulfated Non-gelling polysaccharides	*O*-acylation	Antioxidants	[53]
		Aminated fucoidans	Cancer treatment	[54,55]
Laminarans (Brown Algae)	Non-gelling polysaccharides	*O*-acylation	Antimicrobial activity	[56]
Alginates (Brown Algae)	Gelling properties (depending on Ca^2+^ concentration)	Amidation of carboxylic functions	Resistance towards alginate lyases	[57]
Ulvans (Green Algae)	Gelling properties	*O*-acylation	Surfactants, emulsifier agents, hydrogels	[58,59,60,61]
Chitosan (Crustacean Shell and Shellfish Wastes)	High crystallinity only soluble in acidic aqueous media	Quaternization of amine functions	Carriers for hydrophobic bioactive molecules	[62]
		*N*-acylation	Micellar delivery system	[63]

**Table 2:** Starch modification with different processes.

Process	Acylating Reagent	Solvent and Reagent	Conditions	DS (%)	Reference
Ionic liquid	Acetic anhydride	1-*N*-alkyl-3-methylimidazolium chlorides	130 °C, 4–24 h	20–98	[125]
scCO_2_	Vinyl laurate	Densified CO_2_ + NaOAc	100 °C, 8 MPa, 1.5 h	67	[126]
		Densified CO_2_ + Na_2_SO_4_	100 °C, 8 MPa, 1.5 h	81	[126]
Ball milling	Lauric acid	K_2_CO_3_	80–40 °C, 2–0.5 h	1.48–4.12	[127]
Enzymatic	lauric acid	NaOH (pH 6) + 1% lipase	60 °C, 24 h	3.3–15.1	[128]
Microwaves	Maleic anhydride	-	450 W, 1–5 min	0.2–9.8	[129]

## Data Availability

This review is based on already published results in scientific literature and does not refer to confidential results.
